# Acute myeloid leukemia cells adhere to bone marrow and acquire chemoresistance by downregulating UNC5B expression

**DOI:** 10.3389/fonc.2024.1394443

**Published:** 2024-09-24

**Authors:** Teng Teng, Liping Ren, Jilong Xiao, Zhiyu Shi, Lanbo Li, Chunhong Song

**Affiliations:** ^1^ Shandong Key Laboratory of Traditional Chinese Medicine and Stress Injury, Department of Laboratory Animal Center, Central Hospital Affiliated to Shandong First Medical University, Jinan, Shandong, China; ^2^ Shandong Academy of Occupational Health and Occupational Medicine, Shandong First Medical University & Shandong Academy of Medical Sciences, Jinan, Shandong, China; ^3^ Department of Hematology, Qilu Hospital, Cheeloo College of Medicine, Shandong University, Jinan, Shandong, China; ^4^ Innovative Institute of Chinese Medicine and Pharmacy, Shandong University of Traditional Chinese Medicine, Jinan, Shandong, China; ^5^ Laboratory Animal Center, Qilu Hospital, Shandong University, Jinan, Shandong, China

**Keywords:** acute myeloid leukemia, UNC5B, adhesion, proliferation, chemoresistance

## Abstract

Acute myeloid leukemia (AML) is a malignant tumor of the hematological system. Because of its characteristics of recurrence, refractory and chemoresistance, new therapeutic targets need to be identified. Adhesion and proliferation are characteristics of AML cells, and critical steps in inducing chemotherapy resistance. In this study, we reported that UNC5B inhibits AML cell bone marrow adhesion, inhibits AML cell proliferation and increases sensitivity to chemotherapy. Mechanistically, RNA sequencing (RNA-seq) and experimental results revealed that overexpression of UNC5B inhibits adhesion and proliferation signaling pathways and inhibits the expression of MPZL1, CLDN23, IGF2 and WNT7B. In conclusion, our findings suggest that UNC5B serves as a prognostic indicator and a potential therapeutic target for AML.

## 1 Introduction

Acute myeloid leukemia (AML) is a heterogeneous malignant tumor involving myeloid hematopoietic stem cells (1). Nearly 80,000 people die of AML each year worldwide, and this number continues to increase (2). Patients with relapsed or refractory AML have a very poor prognosis, with complete response (CR) rates ranging from 10% to 30% for patients who relapse within 12 months (3). The diagnostic criteria for refractory AML include: newly diagnosed AML patients who have failed two courses of standard treatment; AML patients who relapse within 12 months after achieving CR and undergoing consolidation and intensive treatment; AML patients who relapse after 12 months and have failed conventional chemotherapy; and AML patients with two or more relapse. Most AML patients achieve CR after initial treatment, during which most AML cells are cleared. However, a small number of drug-resistant cells are not affected and continue to proliferate (4, 5), thus, measurable residual disease (MRD) involving local cells in the bone marrow is a key predictor of refractory or recurrent disease. The pathogenesis of AML has been studied, but further studies are needed to clarify why residual AML cells are present in the bone marrow. Most patients who achieve a CR after first chemotherapy ultimately experience relapse; thus, only a small proportion of patients achieve a second CR after chemotherapy (6), and the long-term disease-free survival rate of patients with AML is only 30%-40% (7). The standard treatment for AML includes long-term combination chemotherapy with cytarabine (Ara-C) and anthracyclines drugs (8), but there is no standard treatment for patients with most types of relapsed or refractory AML; moreover, with increasing age and drug resistance, it becomes more difficult to achieve a secondary CR (9). The adhesion of tumor cells is one of the main reasons for tumor drug resistance. AML cells bind closely to osteoblasts through a series of adhesion molecules, AML cells will be “homing” and become hidden within the bone marrow microenvironment (10).

UNC-5 homologue B (UNC5B) is an important type I transmembrane protein that can play an important role in a variety of biological functions, such as neurogenesis, inflammatory response mediation, tumorigenesis and progression (11), and is also an important adhesion molecule. UNC5B participates in the development of different kinds of cancers, such as ovarian, bladder, and colorectal cancers, by regulating related signaling pathways (12, 13). However, there are currently few reports of its relation and regulatory mechanism to AML. Ntn1 is a ligand of Unc5b, and Ntn1 has been shown to be overexpressed in cutaneous squamous cell carcinoma and to promote tumorigenesis and Epithelial-Mesenchymal Transition (EMT). Upon Ntn1 or Unc5b knockout, EMT is inhibited, and cell migration is suppressed (14). Similarly, NTN1 is upregulated in endometrial cancer, and targeting NTN1 can inhibit tumor progression in endometrial cancer and inhibit EMT (15). NTN1 binding to its receptor UNC5B induces AML cells to resist apoptosis, and the antiapoptotic effect of NTN1 is blocked by UNC5B knockdown, confirming that UNC5B plays an important role as a receptor for NTN1 (16). Thus, with a clear understanding of how altering the expression of the NTN1 receptor UNC5B can inhibit AML cell adhesion and proliferation and increase sensitivity to drugs, the NTN1-UNC5B axis could become an emerging therapeutic target.

In this study, we found that the expression of UNC5B is downregulated in AML samples vs. normal controls; overexpression of UNC5B inhibited the adhesion and proliferation of AML cells. Our study showed that AML cells can adapt to changes in the bone marrow microenvironment. This study may provide a theoretical basis for understanding the mechanism by which AML cells adapt to the bone marrow microenvironment and provide some help for addressing AML drug resistance.

## 2 Materials and methods

### 2.1 Materials and reagent

Fetal bovine serum, cell culture medium 1640, DMEM were obtained from Gibco and purchased from Thermo Scientific (USA). The lentivirus was purchased from Miaoling Biology (China). Experimental animals were purchased from Beijing Vital River Laboratory Animal Technology (China). The primers used were purchased from Keyybio (China). A Cell Counting Kit-8 was purchased from Vazyme Biotech (China). TRIzol was purchased from Thermo Scientific (USA). Annexin V-FITC/PI double-staining cell apoptosis detection kit was purchased from Bestbio (China). RT-qPCR reagent was purchased from ACCURATE BIOTECHNOLOGY(HUNAN) CO., LTD (China). All the above reagents were used according to the manufacturer’s instructions. RNA-seq was performed by Shanghai Biotechnology Corporation.

### 2.2 Patients involved in this study

Samples and data were collected from AML patients with newly diagnosis and healthy donors at Qilu Hospital of Shandong University. All relevant procedures for patient sample collection were approved by the Research and Ethics Committee of Qilu Hospital of Shandong University, and all patients who provided clinical samples signed informed consent forms. The patient’s information can be found in **Supplementary Table S1**. All the procedures were performed in accordance with the 1964 Declaration of Helsinki principles and its later amendments or comparable ethical standards.

### 2.3 Cell culture

Human acute myeloid leukemia cells (THP-1 and Molm13) were obtained from China National Collection of Authenticated Cell Cultures, human umbilical vein endothelial cell HUVEC was obtained from Shandong University of Physiology Teaching and Research Office; human embryonic kidney cell HEK-293T was obtained from China National Collection of Authenticated Cell Cultures, *KMT2A::MLLT3*-GFP cells were donated by the Institute of Hematology, Chinese Academy of Medical Sciences. The cells were cultured in an incubator with a continuous supply of 5% CO2 under humidified conditions (75-80% relative humidity). THP-1 cells, Molm13 cells and HUVECs were grown in RPMI-1640 culture medium supplemented with 10% fetal bovine serum (FBS), and HEK293T cells were grown in DMEM supplemented with 10% FBS. These cell lines were identified by short tandem repeats (STR) and stored in the laboratory of Hematology, Qilu Hospital, Shandong University. All cell lines have been authenticated using STR within the last three years.

### 2.4 RNA isolation and real-time quantitative PCR

Total RNA was extracted using the TRIzol method according to the manufacturer’s instructions and reverse-transcribed into cDNA using SYBR^®^ Green Premix Pro Taq HS. Quantitative real-time PCR was performed using TB Green Premix Ex Taq™ II on a Light Cycler 480 II (Roche). GAPDH was used as an endogenous control. The data were analyzed using the 2^-△△CT^ method. The primers used for RT−qPCR were as follows: UNC5B for human: forward: 5′′-GTCGGACACTGCCAACTATAC-3′, reverse:5′-CCGCCATTCACGTAGACGAT-3′. GAPDH for human: forward: 5′- TGCACCACCAACTGCTTAGC-3′, reverse: 5′-GGCATGGACTGTGGTCATGAG-3′. MPZL1 for human: forward: 5′-GGCAGAGAATCCTCACCAGT-3′, reverse: 5′-GTGTGAGCAGCTTCCTTCAG-3′, CLDN23 for human: forward: 5′-AGTGGACGTGGAGTTGTACC-3′, reverse: 5′-AGCGAGGTGACCATGAGTG-3′, IGF2 for human: forward: 5′- ATGACACCTGGAAGCAGTCC -3′, reverse: 5′-TGGGTGGGTAGAGCAATCAG-3′, WNT7B for human: forward: 5′-GCAGTGCAACTGCAAATTCC-3′, reverse: 5′-CACTTGCAGGTGAAGACCTC-3′.

### 2.5 Animals

6-8 week-old male C57BL/6 mice were selected, with 5-6 mice in each group. All animals were kept in specific pathogen-free (SPF) level animal rooms at the Animal Center of Qilu Hospital, Shandong University. All animal experiments were approved by and conducted under the guidance of the Animal Ethics Committee of Shandong University Qilu Hospital.

For the AML mice model, *KMT2A::MLLT3* cells with lentivirus transfection (Vector & Over-expression(OE)-UNC5B) were injected into C57BL/6 mice via tail vein (1 × 10^7^ cells per mouse for homing analysis and 5 × 10^6^ cells per mouse for before and after treatment analysis). Homing analysis was performed after 16 hours, before and after treatment analysis was performed after 7 days. We injected Ara-C and doxorubicin (DOX) intraperitoneally for three days after day 7. On day 11, we harvested the unilateral femur bone marrow cells and splenocytes. The percentage of tumor burden was analyzed by flow cytometry.

### 2.6 Lentivirus packaging and infection

For the tool cell line HEK-293T, PMD2g and PspaX2 packaging plasmids, the vector plasmid pLV3-CMV-UNC5B-3×FLAG-mCherry-Puro, and Lipofectamine 2000 were used for virus packaging. The viruses were transduced, and the collected supernatant was centrifuged and filtered through a 0.45 µm filter. For lentivirus infection, UNC5B virus was added directly to the cells. Screening was performed with 1μg/mL of puromycin.

### 2.7 Analysis of adhesion, proliferation, chemosensitivity and apoptosis

For the detection of cell adhesion, a total of 5×10^5^ AML cells were seeded in 6-well plates covered with HUVECs. After direct contact for 6 h, the culture supernatant was removed, 2 mL of PBS was slowly added along the edge of the plate, the nonadherent cells were washed off, and the cells were washed again. The PBS was removed, a fluorescence microscope was used to view the cells, and the number of mCherry-positive cells adhering to the HUVECs was counted.

UNC5B was overexpressed in AML cells, Molm13 and THP-1 cells were seeded on 96-well plates, and the proliferative ability of the control group was examined via a CCK-8 kit, after which the proliferation of the cells was measured at 0, 24, 48, 72, and 96 h. In the CCK-8 assay, WST-8 is the key chemical. It is reduced by NAD+ to a water-soluble yellow formazan product in the presence of viable cells and active metabolism. The more live cells present, the more formazan is produced, resulting in a darker color. The viability of AML cells treated with Ara-C and DOX were detected via CCK-8 assays. We have set the drug concentrations for Ara-C and DOX based on previous research (17). Molm13 and THP-1 cells were seeded on 96-well plates, Molm13 and THP-1 cells were treated with Ara-C and DOX doses of 0.01 μM, 0.05 μM, 0.25 μM, 1.25 μM, and 6.25 μM for each drug. The absorbance at 450/630nm was measured with a microplate reader.

The percentage of apoptotic cells was measured using an Annexin V-FITC/PI double-staining cell apoptosis detection kit. Q2: (AnnexinV+FITC)+/PI+; Q3: (AnnexinV-FITC)+/PI-; Q4: (AnnexinV-FITC)-/PI-. Molm13 and THP-1 cells were stained according to the kit manufacturer’s protocol and analyzed using flow cytometry. The apoptotic rate was defined as the percentage of early and late apoptotic cells among all cells in the sample (Q2 and Q3, respectively).

### 2.8 RNA-seq

For RNA-seq, the VAHTS Universal V6 RNA-seq Library Prep Kit for Illumina^®^ (Vazyme, #NR604-01) was used to establish the library. The cells in each group were sequenced with an Illumina HiSeq 2000 platform, and the length of the single-end sequences ranged from 200-300 bp. Sequence reads were compared with version 38 of the human genome (GRCH38) using a standard Illumina sequence analysis pipeline.

### 2.9 scRNA-seq data processing and GEPIA

In brief, the gene barcode count matrix for the GSE178912 dataset was analyzed using the Seurat R software package (version 4.0.2). The GSE178912 dataset contains single-cell RNA-Seq data of residual AML cells purified from patient-derived xenograft bone marrow, with or without treatment using venetoclax plus cytarabine. Cells with > 200 genes and < 10% mitochondrial gene expression were screened via downstream analysis. After the samples were converted to Seurat, the combined Seurat data were normalized and scaled by regression of UMI count and mitochondrial gene percentage. In terms of dimension reduction, the Find Variable Genes function was used to identify the most variable genes. PCA was subsequently used for dimensionality reduction, and the UMAP graph was generated by the Run UMAP Seurat function (Seurat version 3.1.3). GEPIA (Gene Expression Profiling Interactive Analysis) is an online bioinformatics tool that uses RNA sequencing data from 9,736 tumor tissues and 8,587 normal tissues in the TCGA and GTEx databases. It retrieves gene expression values in various tumor samples, calculates gene expression levels in specific tumors, and analyzes the relationship between genes and tumor prognosis, as well as gene co-expression. (http://gepia.cancer-pku.cn/).

### 2.10 Statistical analysis

The sample size was chosen to be n ≥ 3. The data were analyzed and are expressed as the medium ± range, mean ± standard deviation. Unpaired t-test were used to analyzed the two groups as indicated; two-way ANOVA were used to compare the inter- and intragroup differences in two factors between two groups as indicated. All the above data were analyzed and plotted using GraphPad Prism 9.0 (La Jolla, CA), and P < 0.05 indicated statistical significance. Flow analysis was performed using FlowJo software (TreeStar, USA) and R software (version 4.2.3). The workflow for animal experiments and Graphical Abstract were illustrated by Figdraw.

## 3 Results

### 3.1 UNC5B expression is related to AML and its drug resistance phenotype

**Figure 1:**
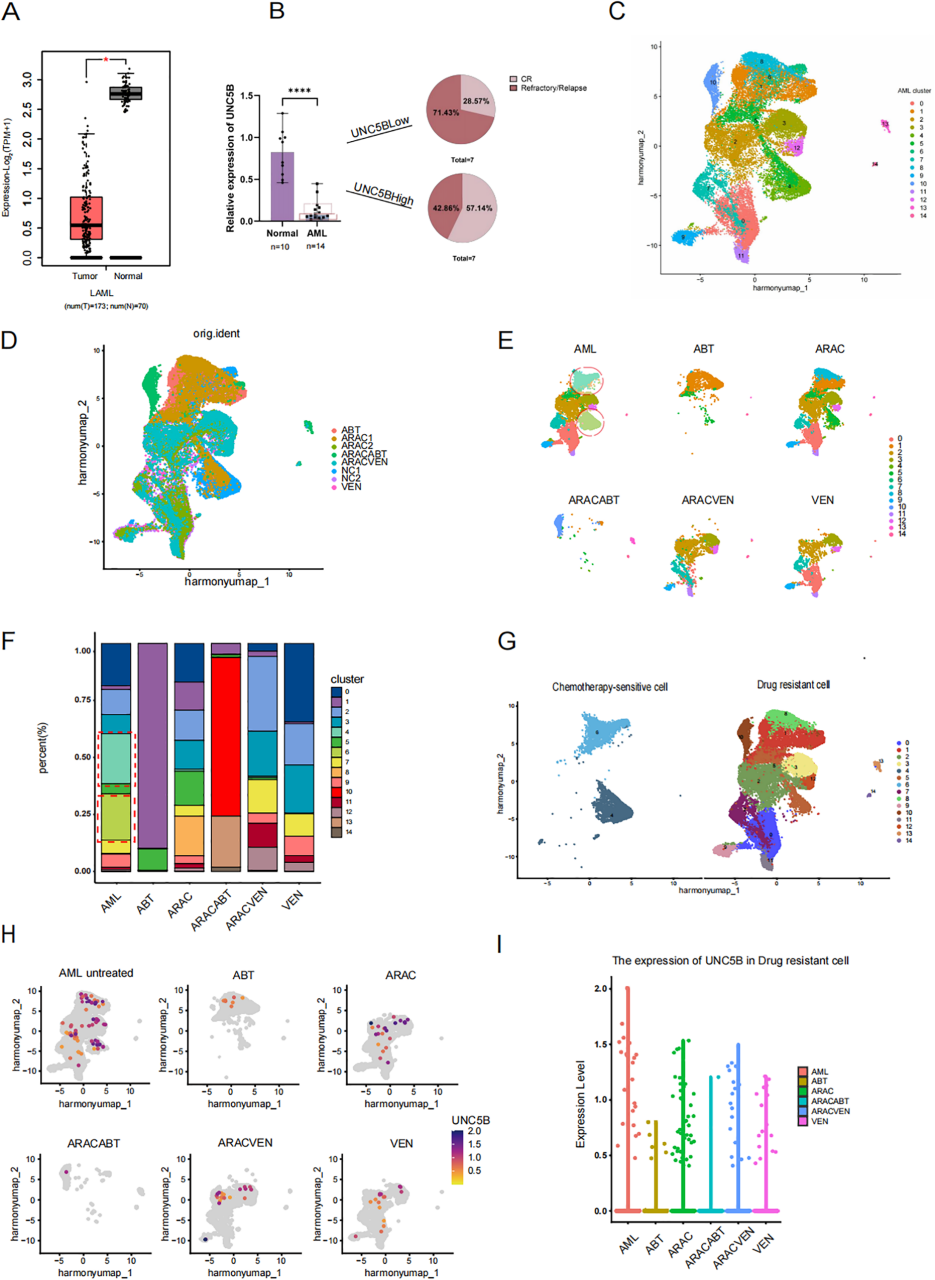
Low expression of UNC5B is associated with refractory and relapse of acute myeloid leukemia. **(A)** The Gene Expression Profiling Interactive Analysis (GEPIA) data showed that the expression of UNC5B in Acute myeloid leukemia (AML) patients with lower expression than healthy donors *P<0.05. **(B)** qRT−PCR analysis of UNC5B mRNA expression in AML patients (n=14) and healthy donors (n=10). Data are presented as medium ± range. ****P < 0.0001, t-test. **(C)** Divide AML cells into 15 clusters, including both treated and untreated groups. **(D)** AML single-cell data of cells before and after treatments. **(E)** AML single-cell data of cells and each different treatment methods. ABT199: Venetoclax; ARAC: Cytarabine; ARACABT: combination therapy of Cytarabine and Venetoclax; ARACVEN: combination therapy of Cytarabine and Venetoclax; VEN: Venetoclax. **(F)** The cluster of each cell subgroup in each group of AML. **(G)** The definition of chemotherapy-sensitive cells and chemotherapy-resistant cells. **(H)** The expression of UNC5B in various cells groups. **(I)** The expression of UNC5B in drug-resistant cells.

UNC5B expression was found to be lower in AML patients than in healthy donors according to analysis of the GEPIA database (**Figure 1A**). Moreover, to verify this conclusion, we collected information from AML patients (**Supplementary Table S1**) and healthy donors to analyzed the relationship between UNC5B expression levels in both groups. The expression levels of UNC5B were significantly lower in the AML group than in the healthy control group, which corresponds to the GEPIA results. An analysis of patient clinical data revealed an association between UNC5B expression and relapse or CR rate: low UNC5B expression indicated a higher relapse rate in AML patients (**Figure 1B**).

First, we searched the Gene Expression Omnibus (GEO) database and selected a dataset of AML patients, including those who underwent different treatment methods. Such as single-agent application of Cytarabine and Venetoclax; or combination therapy of Cytarabine and Venetoclax (**Supplementary Table S2**). The following results below are based on the GEO dataset. Based on this, we used R language to analyze AML patients included in the dataset before and after treatment to examine UNC5B expression and its relationship with drug resistance in AML cells. The growth protocol of the dataset we have selected from GEO is the process of isolating and purifying human AML cells, xenotransplanting them into 8-week-old NSG mice for amplification, the human AML cells from the mice’s bone marrow were isolated and purified to conduct single-cell sequencing. The lack of immune cells in NSG mice made this data more accurately to demonstrate the effects of various drugs on leukemia cells. We conducted single-cell analysis on the sorted leukemia cells from the dataset, we divide AML cells which have received different treatments and who have not received treatment into 15 AML clusters (**Figure 1C**). Meanwhile, to verify that these clusters are all AML cells, we used the same marker, CD33, as the source data and verified that these clusters were all indeed leukemia cells (**Supplementary Figure S1**). The treatment types in this dataset include single-agent application of Cytarabine and Venetoclax; or combination therapy of Cytarabine and Venetoclax (**Figure 1D**). By observed the changes in the cell proportion in each AML clusters before and after chemotherapy drugs, we found that the proportion of two clusters, clusters 4 and 6, was significantly decreased after treatment (**Figures 1E, F**). Therefore, we consider that these two groups of cells, which are significantly reduced after chemotherapy, will be called chemotherapy-sensitive cells, while the other cells were designated as drug-resistant cell subpopulations (**Figure 1G**). To further investigate the role of UNC5B in AML, we analyzed the expression levels of UNC5B in six groups of AML cells and found that the expression of UNC5B in the chemotherapy group were lower than untreated group (**Figure 1H**). Moreover, we extracted drug-resistant cells in the untreated group and compared the expression of UNC5B in the drug-resistant cells in other chemotherapy groups. For the drug-resistant cells, the expression of UNC5B was significantly reduced after treatment (**Figure 1I**). These findings suggested that decreased expression of UNC5B may enhance the drug resistance of resistant cells in AML cells. Taken together, these results support our conclusion that UNC5B expression is low in AML and is associated with drug sensitivity. So, overexpression of UNC5B may increase chemotherapy sensitivity.

### 3.2 Over-expression of UNC5B suppresses AML cell homing and promotes chemotherapy sensitivity

**Figure 2:**
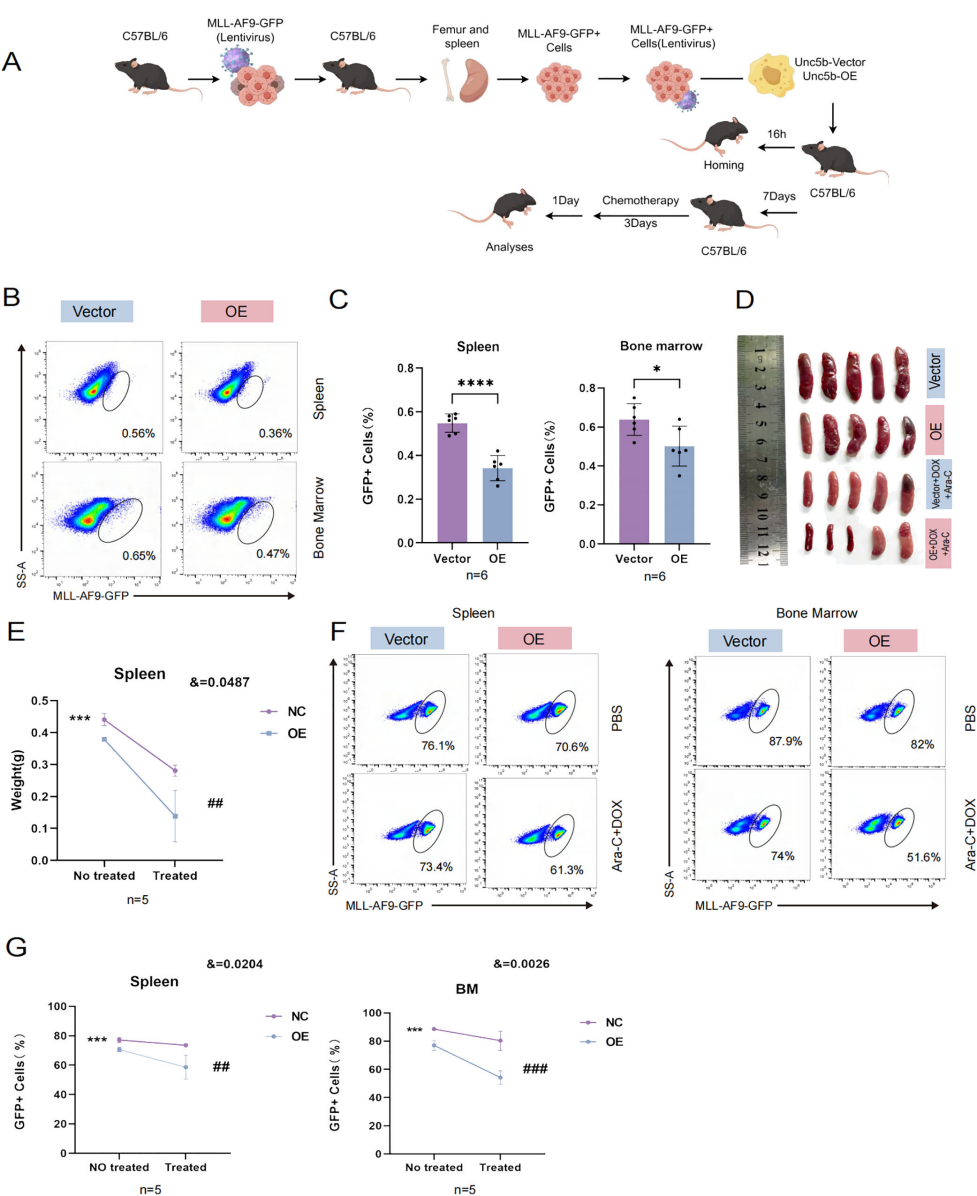
UNC5B inhibited homing and improved chemotherapy sensitivity of AML cells. **(A)** Schematic outline of the experimental strategy used to establish AML homografts from *KMT2A::MLLT3* leukemia cells. **(B, C)** Homing of *KMT2A::MLLT3* cells in the spleen and BM are shown and summarized (n=6). Data are presented as mean ± SD. *P < 0.05, ****P < 0.0001. **(D, E)** Spleen image and weight indices of mice treated with Cytarabine (Ara-C) and doxorubicin (DOX) at the end of the experiment (n=5). Data are presented as mean ± SD. ***P < 0.001, vs. the NC group not treated; ##, P < 0.01, vs. the NC group treated with chemotherapy; &, significant interaction effect; two-way ANOVA. **(F, G)** The tumor burden values of *KMT2A::MLLT3* cells in the spleen and bone marrow (BM) are shown and summarized (n=5). Spleen: Data are presented as mean ± SD. ***P < 0.001, vs. the NC group not treated; ##, P < 0.01, vs. the NC group treated with chemotherapy; &, significant interaction effect; two-way ANOVA. BM: Data are presented as mean ± SD. ***P < 0.001, vs. the NC group not treated; ###, P < 0.001, vs. the NC group treated with chemotherapy; &, significant interaction effect; two-way ANOVA. n ≥ 5, mean ± SD values are shown for **(C, E, G)**.

In order to prove whether UNC5B is related to chemotherapy sensitivity of AML, we used *in vitro* and *in vivo* experiments to verify. *In vivo* experiment, we constructed a mouse model of *KMT2A::MLLT3* leukemia. We isolated spleens from *KMT2A::MLLT3* mice and obtained leukemia cells. These cells were then transfected with UNC5B and control vectors *in vitro.* After *KMT2A::MLLT3* leukemia cells were transfected with UNC5B lentivirus, they were injected into C57BL/6 mice via the tail vein. Bone marrow from the homing model was obtained at 16 h, and a before and after treatment model with DOX + Ara-C injection was used to assess whether UNC5B influenced chemosensitivity in AML cells (**Figure 2A**). We performed *in vivo* data analysis of the homing model and found that UNC5B overexpression decreased the percentage of GFP-positive AML cells in the bone marrow and spleen of mice, indicating that UNC5B overexpression suppressed the homing of AML cells to the bone marrow and spleen (**Figures 2B, C**).

Next, we used DOX in combination with Ara-C as the chemotherapeutic regimen and assessed how UNC5B expression affected chemosensitivity *in vivo*. We found that the overexpression of UNC5B significantly increased the sensitivity of AML model mice to chemotherapeutic agents, and the size and weight of the spleen significantly decreased (**Figures 2D, E**). The tumor burden was reduced in the bone marrow and spleens of AML mice which were treated with chemotherapeutic drugs (**Figures 2F, G**). These results might indicate that UNC5B improved chemosensitivity in AML cells *in vivo*.

### 3.3 UNC5B improves chemosensitivity by inhibiting AML cell adhesion, proliferation

**Figure 3:**
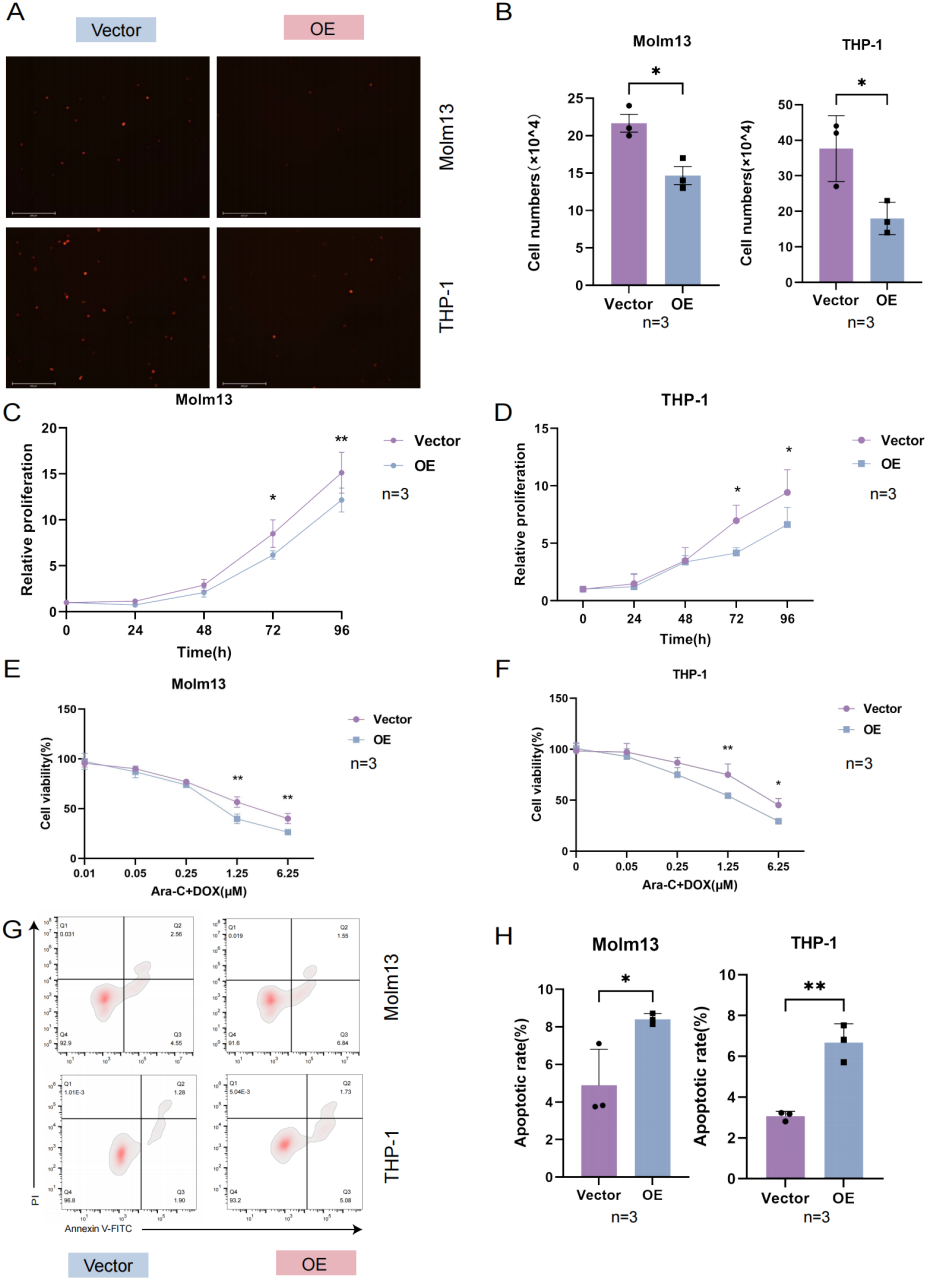
UNC5B affected the adhesion, proliferation and chemosensitivity of AML cells. **(A, B)** Images and corresponding statistical results showed that UNC5B overexpression inhibited the adhesion of AML cells (n=3). Data are presented as mean ± SD. *P < 0.05, t-test. **(C, D)** CCK8 assays showed reduced proliferation in UNC5B overexpression in Molm13 and THP-1 cells (n=3). Data are presented as mean ± SD. *P < 0.05, **P < 0.01, t-test. **(E, F)** The CCK-8 assays showed UNC5B improved the chemosensitivity of Molm13 and THP-1 cells (n=3). Data are presented as mean ± SD. *P < 0.05, **P < 0.01, two-way ANOVA. **(G, H)** Flow cytometry analysis showed apoptosis of UNC5B overexpression in Molm13 and THP-1 cells with low-dose drug treatment (n=3). Data are presented as mean ± SD. *P < 0.05, **P < 0.01, t-test. n ≥ 3, mean ± SD values are shown for **(A–H)**.

Cell adhesion refers to the use of specific surface adhesion molecules (AMs) attached to adjacent cells or the cell matrix. It is a key link in the occurrence and development of AML. At present, an increasing number of studies have focused on leukemia-targeted adhesion (16). An essential characteristic of tumor cells is that they continually proliferate and affect cancer progression (17). Strategies for targeting and inhibiting the proliferation of tumor cells are of great significance in inhibiting the development of AML. To further clarify the mechanism of drug sensitivity, we performed further experiments. By coculturing with HUVECs, we found that the adhesion of AML cells in the UNC5B-overexpressing group was reduced compared to the control group, which is consistent with the results of our *in vivo* experiments (**Figures 3A, B**). Next, we performed a proliferation assay to examine the proliferative capacity of 0-96 h Molm13 cells and THP-1 cells by a CCK-8 assay. Moreover, the ability of cells to proliferate was significantly lower in the UNC5B-overexpressing group than in the control group, supporting our view that UNC5B overexpression suppressed the adhesion and proliferation of cells (**Figures 3C, D**). To further verify the role of UNC5B in chemosensitivity, we used Ara-C and DOX to treat Molm13 and THP-1 cells. The viability rate of Ara-C and DOX-treated cells in the overexpressing group was lower than that of PBS-treated controls under Ara-C and DOX drug treatment, thus, UNC5B overexpression improved the chemosensitivity of AML cells (**Figures 3E, F**). Afterwards, we tested the apoptotic ability of AML cells under low-dose drug treatment and found that after treatment with low-dose chemotherapy drugs, the apoptosis of AML cells overexpressing UNC5B increased (**Figures 3G, H**). Finally, we conducted an apoptosis assay to measure the apoptotic rate of Molm13 and THP-1 cells without drugs using flow cytometry. The results showed that there was no significant difference in the apoptosis rate between the UNC5B overexpressing group and the control group (**Supplementary Figure S3**). Overexpression of UNC5B can inhibit cell adhesion and proliferation, and reduce cell activity under the pressure of chemotherapy, which making leukemia cells more sensitive to the effects of chemotherapy.

### 3.4 The underlying molecules that mediated by UNC5B in AML adhesion and proliferation

**Figure 4:**
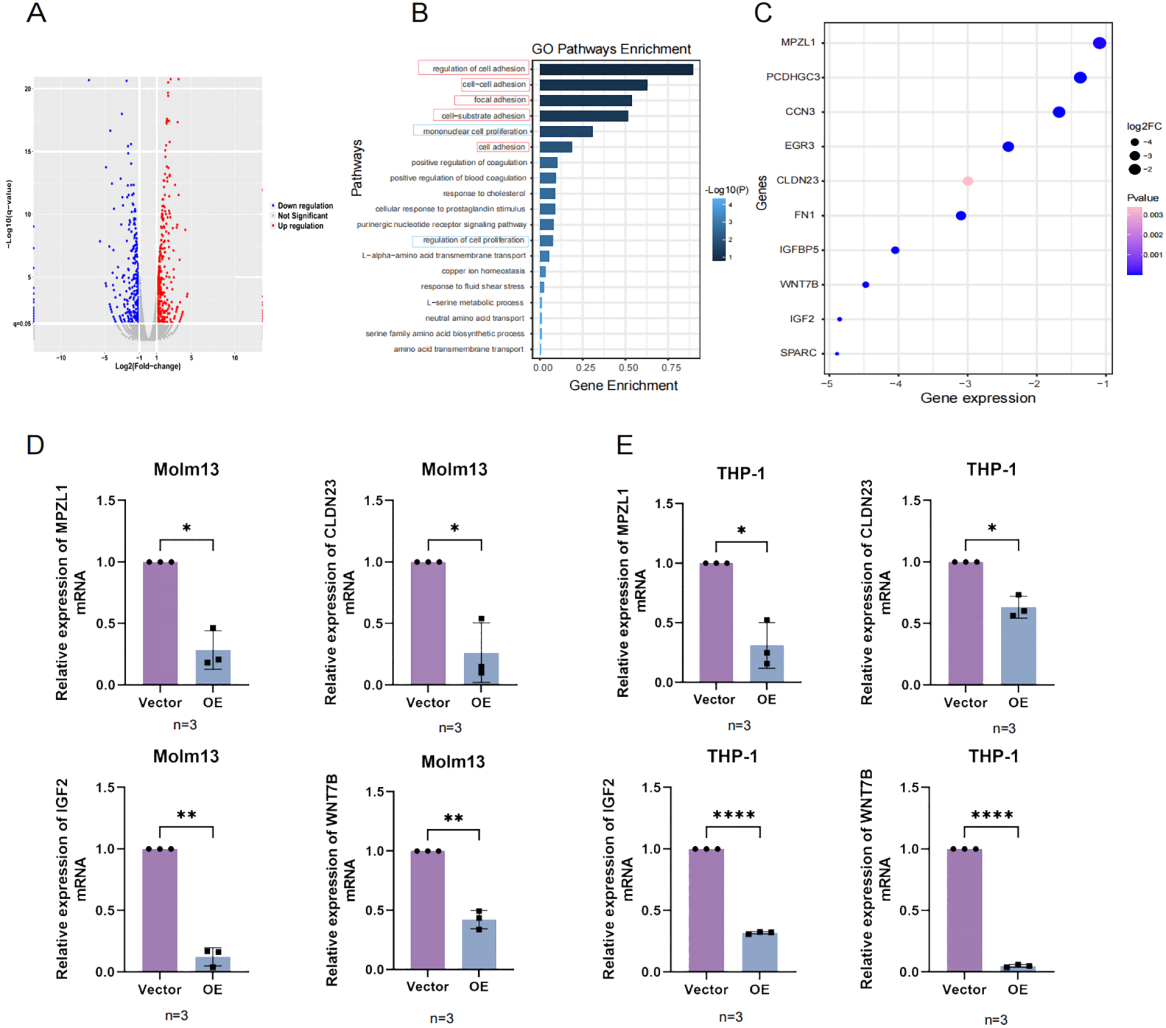
The RNA-seq results confirmed that UNC5B is associated with AML cell adhesion and proliferation. **(A)** The volcano plot results indicated the upregulation and downregulation of genes in the overexpression group and vector group. **(B)** Gene Ontology (GO) enrichment pathways related to adhesion and proliferation. **(C)** Molecular genes expression related to adhesion and proliferation. **(D, E)** The expression of MPZL1, CLDN23, IGF2, and WNT7B in the overexpression and control groups of Molm13 and THP-1 cells (n=3). Data are presented as mean ± SD. *P < 0.05, **P < 0.01, ****P<0.0001, t-test. n ≥ 3, mean ± SD values are shown for (D, E).

To verify whether UNC5B affects adhesion and proliferation-related genes, we performed an RNA-seq analysis. After overexpression of UNC5B, the expression levels of many genes were altered (**Figure 4A**). Based on these differentially expressed genes, we conducted GO pathway enrichment analysis and found that the main altered pathways were related to adhesion and proliferation related (**Figure 4B**). Moreover, we found that the adhesion and proliferation molecules were significantly altered in these pathways. We evaluated molecules in adhesion- and proliferation-related pathways, such as myelin Protein Zero-Like Protein (MPZL1), Claudin-23 (CLDN23), Wnt Family Member 7B (WNT7B), and Insulin-like growth factor 2 (IGF2), and found that they significantly decreased after overexpressing UNC5B (**Figure 4C**). We then analyzed AML and normal samples from the TCGA and GTEx databases, focusing on the expression levels of four genes. Our analysis revealed that these genes were expressed at higher levels in AML patients compared to normal samples (**Supplementary Figure S4**). Subsequently, we validated the expression of these molecules using RT−qPCR experiments. The results confirmed that UNC5B could affect the development of AML by inhibited the expression of adhesion and proliferation genes of AML cells (**Figures 4D, E**).

## 4 Discussion

AML is a hematological malignant disease caused by the abnormal proliferation of immature myeloid progenitor cells, and the local bone marrow microenvironment is one of the main factors involved in AML pathogenesis (18). Because of their homing and migration abilities and other biological characteristics, AML cells rapidly home to the bone marrow and “hijack” normal hematopoietic stem cell niche signals to promote the proliferation of leukemia-initiating cells and regulate their adhesion and movement (19). *In vivo* transplantation of mice after treatment with VLA4 antibody reduced the homing of bone marrow hematopoietic progenitors, and treatment with VCAM-1 antibody yielded similar results; it inhibited the homing of hematopoietic progenitor cells to the bone marrow (20). Adhesion molecules not only act as bridges between cells but also transduce signals when they are activated as receptors, thus promoting the occurrence and development of tumors (21). The NTN1(Ntn1)-UNC5B(Unc5b) pathway was recently identified as an important molecular system associated with tumor adhesion (14, 15), but whether this pathway mediates bone marrow adhesion in suspended cells has not been reported. Our study confirmed that AML cells promote their own bone marrow adhesion and drug resistance by downregulating UNC5B expression, which provides a mechanistic basis for the treatment of AML through the inhibition of bone marrow adhesion. We were excited to discover that a clinical trial is currently underway in AML patients, evaluating the safety and clinical activity of the anti-NTN1 antibody NP137 in combination with Azacitidine and Venetoclax for refractory AML patients (ClinicalTrials.gov identifier NCT06150040). We hope that this clinical trial will yield positive results, providing more treatment options for AML patients and contributing to further research on chemotherapy resistance in AML.

UNC5B is closely related to the genesis of many tumors. Among colorectal cancer patients, those in the UNC5B-Low expression group had a worse prognosis than those in the UNC5B-high-expression group, and low UNC5B expression was an independent risk factor for postoperative recurrence in patients with different tumor stages and categories (13). In contrast, UNC5B is highly expressed in ovarian and breast cancers, and knockdown UNC5B experiments in ovarian cancer showed that it affects cancer cell proliferation and migration (22, 23). It has also been found to be a potential antitumor target in bladder cancer; overexpression of UNC5B can significantly inhibit the proliferation and metastasis of bladder cancer cells (24). UNC5B interacts with NTN1 in B-ALL, and after knocking down UNC5B in B-ALL cells, NTN1 inhibits B-ALL cell apoptosis through abnormal activation of the FAK-MAPK pathway by the UNC5B receptor (25). In a previous study, NTN1 was shown to regulate the FAK-Akt-NF-κB signaling pathway through UNC5B and participate in the occurrence and development of AML, regulating the proliferation and apoptosis of AML cells. We found that NTN1 stimulation *in vitro* increased cell apoptosis. However, they found that the apoptosis of AML cells was significantly inhibited when 50ng/ml of NTN1 was applied, downregulating UNC5B expression alone does not alter cell apoptosis (16). In endothelial cells, UNC5B induces apoptosis in the absence of NTN1, but this apoptosis is blocked when NTN1 binds (26). Similarly, our experimental results align with previous findings, showing that overexpression of UNC5B alone does not significantly affect AML cell apoptosis. This highlights the distinct functions of UNC5B in different cell types and underscores its importance. We consider that NTN1 is relatively abundant *in vivo*, and UNC5B primarily plays an adhesive function, which limits its impact on cell apoptosis. While, no research has shown how UNC5B affects the homing and enrichment of AML cells. We found that UNC5B enhances cell adhesion via NTN1, creating a conducive homing environment. UNC5B can regulate the proliferation and adhesion of AML cells and affects their sensitivity to chemotherapy *in vitro* and *in vivo.* These findings suggest that UNC5B has a role in the development of AML. The AML cells would adapt to the bone marrow microenvironment by downregulating UNC5B, ultimately leading to chemoresistance in AML. Using the Bloodspot database, we observed that UNC5B expression was elevated in certain AML cells with abnormal karyotypes compared to normal progenitor cells. Additionally, the GEPIA database revealed that UNC5B expression is generally lower in AML patients than in healthy individuals. These findings indicate that UNC5B expression varies among AML patients, likely due to the inherent heterogeneity. Multiple factors like geographic region, ethnicity, gender, and age may influence UNC5B expression levels. Our study has limitations due to the small sample size and diversity of patient subtypes. However, the limited sample size prevented us from definitively identifying UNC5B specificity across different AML subtypes. In future work, we plan to establish cohorts of AML patients with distinct subtypes to further refine the characterization of UNC5B expression levels. We aim to collect more samples to validate our conclusions and further explore the relationship between AML subtypes and UNC5B expression. Our mice model simulates the pathogenesis of human AML by introducing the *KMT2A::MLLT3* fusion gene which had become the most widely used *in vivo* leukemia model (27, 28). *KMT2A::MLLT3* fusion gene, as a carcinogenic factor, can promote AML cells proliferation (29). So we injected *KMT2A::MLLT3* cells into both the control and experimental groups in our animal experiments, and UNC5B-overexpression inhibited cell proliferation on the basis of *KMT2A::MLLT3*. It is worth investigating whether there is an interaction between UNC5B and *KMT2A::MLLT3* in regulating AML cells adhesion and proliferation. Our *in vivo* model used the C57BL/6J mouse model, consistent with previous literature. Additionally, we noted that CD45.1/CD45.2 animals have been used to study AML disease progression. CD45.2 primary hematopoietic precursors were transduced with a retrovirus carrying the *KMT2A::MLLT3* fusion gene. After *in vitro* culture, the transduced cells were injected into lethally irradiated CD45.1 mice via the tail vein to evaluate the leukemia model. We consider that future research should further utilize the CD45.1/CD45.2 animal model to gain a deeper understanding of AML mechanisms.

For proliferation-related gene: IGF2 is a key regulator of cell proliferation. Marine et al. demonstrated its critical role in adrenal carcinoma cells where IGF2 overexpression significantly affects proliferation (30). In anal squamous cell carcinoma (ASCC), cancer-associated fibroblasts secrete IGF2, promoting the proliferation of human ASCC cell lines through paracrine signaling (31). WNT7B, another proliferation-related gene, is part of the WNT family. It has been shown to induce bile duct cell proliferation (32). Joseph’s studies revealed that WNT7B promotes endothelial cell proliferation in a dose-dependent manner both *in vivo* and *in vitro* (33). CLDN23 plays a vital role in tight junctions and cell adhesion. Natalia et al. correlated transcriptomic datasets with enhancer of zeste 2 polycomb repressive complex 2 subunit (EZH2) binding densities and revealed that the CLDN23 gene, which encodes a component of cell-cell adhesion structures, is occupied by EZH2 and has reduced expression in colorectal cancer (CRC) tissue (34). The MPZL1 is another adhesion-related gene. The MPZL1 signaling pathway plays a crucial role in breast cancer metastasis by mediating cell adhesion in HER2+ breast cancer cells (35). Additionally, research indicates that phosphorylated MPZL1 is linked to cell adhesion (36). These four genes are critical for proliferation and adhesion and play important roles in various tumors. According to the report, these four molecules regulated adhesion and proliferation through many signaling pathways, for instance, PI3K-Akt, Wnt/β-Catenin, Rap1 and others (37–39). Meanwhile, the knockout of UNC5B inhibits breast cancer cell proliferation and impairs the activation of the PI3K-Akt pathway (23). These two signaling pathways may relate to the regulation of molecules by UNC5B, more investigation and study are required to support this result.

In conclusion, we found that UNC5B inhibits bone marrow cell adhesion and proliferation in AML and enhances sensitivity to chemotherapy *in vivo* and *in vitro*. Overexpression of UNC5B suppresses signaling pathways and gene expression related to adhesion and proliferation. Our findings suggest that UNC5B could be used as a prognostic marker and a potential therapeutic target for AML.

## Data Availability

The data presented in the study are deposited in the GEO database, accession number GSE277482.
