# Supplementary material for: Acute myeloid leukemia cells adhere to bone marrow and acquire chemoresistance by downregulating UNC5B expression
Source: Front Oncol. 2024 Sep 24;14:1394443. doi: 10.3389/fonc.2024.1394443 (PMC11460579; doi:10.3389/fonc.2024.1394443)

Supplementary Material

1 Supplementary Figures and Table

1.1 Supplementary Table

Basic characteristics of the patients in this study. See Supplementary Table 1 for details.

1.2 Supplementary Figures

Supplementary Figure 1. All detected cell clusters are AML cell populations.

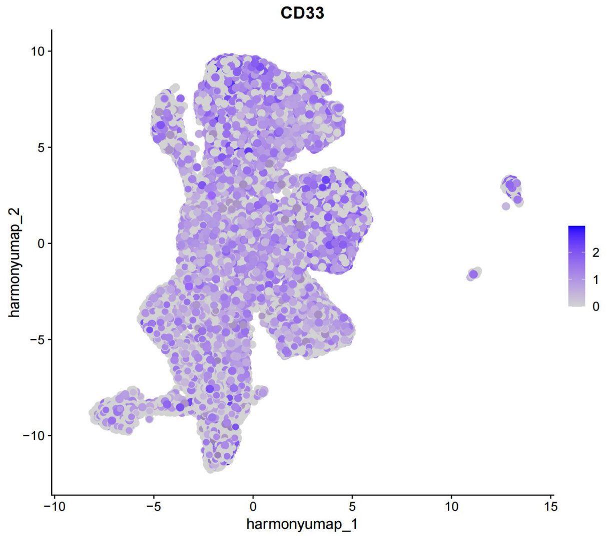

Supplementary Figure 2 . Isolated CD34+ cells from the bone marrow of donors and AML patients to conduct RT-qPCR experiments. **(A)** The proportion of CD34+ cells in donors bone marrow. **(B)** The proportion of CD34+ cells in AML patients bone marrow.

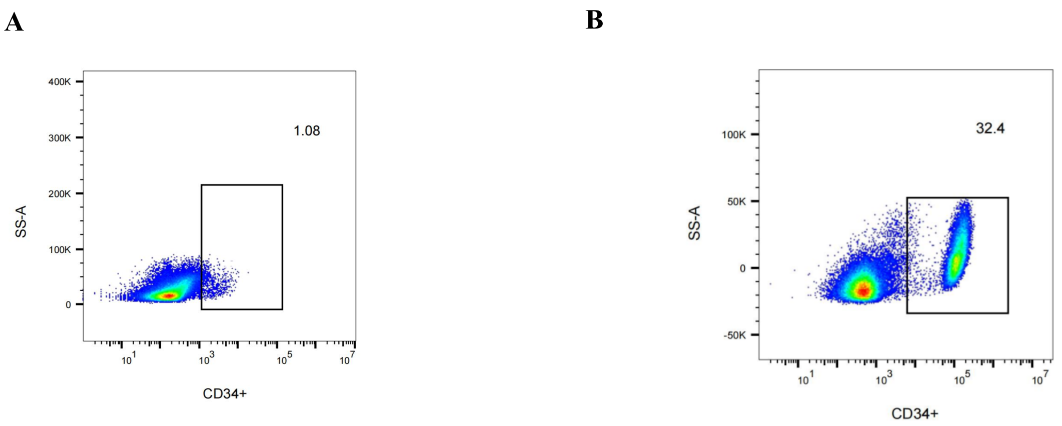

Supplementary Figure 3. **(A, B)** Flow cytometry analys is showed apoptosis of UNC5B overexpression in Molm13 and THP-1 cells **(**n=3**)**.
Data are presented as mean± SD. ns, Not significant.

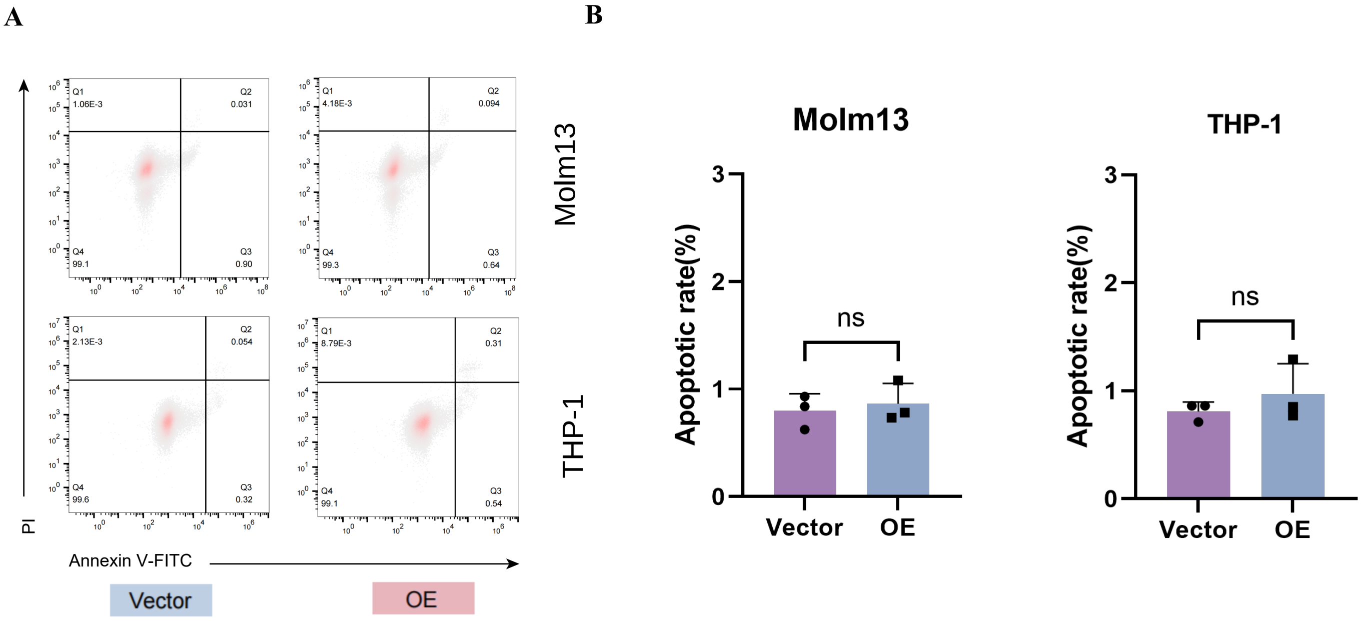

Supplementary Figure 4 . The expression levels of MPZL1, CLDN23, WNT7B and IGF2 were analyzed in the TCGA and GTEx datasets.

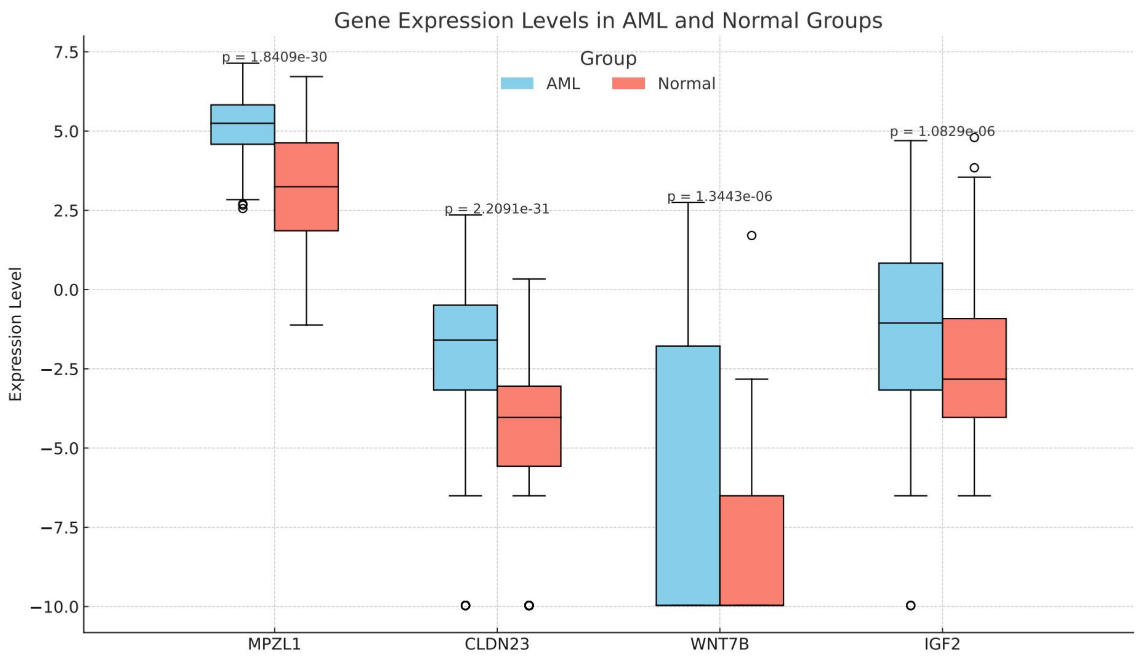

Supplement: Supplementary file 1 [file DataSheet1.pdf]
